# Classification of fetal and adult red blood cells based on hydrodynamic deformation and deep video recognition

**DOI:** 10.1007/s10544-023-00688-6

**Published:** 2023-12-14

**Authors:** Peter Johannes Tejlgaard Kampen, Gustav Ragnar Støttrup-Als, Nicklas Bruun-Andersen, Joachim Secher, Freja Høier, Anne Todsen Hansen, Morten Hanefeld Dziegiel, Anders Nymark Christensen, Kirstine Berg-Sørensen

**Affiliations:** 1 Department of Applied Mathematics and Computer Science, Technical University of Denmark, Kongens Lyngby, Denmark; 2 Department of Health Technology, Technical University of Denmark, Kongens Lyngby, Denmark; 3 Department of Clinical Immunology, University Hospital Copenhagen, Copenhagen, Denmark; 4 Department of Clinical Medicine, Copenhagen University, Copenhagen, Denmark; 5 Kongens Lyngby, Denmark

**Keywords:** Red blood cell, Microfluidic flow cytometry, Deformation, Neural network, SlowFast

## Abstract

Flow based deformation cytometry has shown potential for cell classification. We demonstrate the principle with an injection moulded microfluidic chip from which we capture videos of adult and fetal red blood cells, as they are being deformed in a microfluidic chip. Using a deep neural network - SlowFast - that takes the temporal behavior into account, we are able to discriminate between the cells with high accuracy. The accuracy was larger for adult blood cells than for fetal blood cells. However, no significant difference was observed between donors of the two types.

**Supplementary Information:**

The online version contains supplementary material available at 10.1007/s10544-023-00688-6.

## Introduction

Several blood diseases - or complications - exist, where it is prudent to differentiate between blood cells of fetal and adult origin. The main motivation behind this study is Feto-Maternal Hemorrhage (FMH). It is a common pregnancy complication where the fetus bleeds into the maternal circulation. Several methods of diagnosis currently exist, including the Betke-Kleihauer test and flow cytometry (Linderkamp et al. 1986b). The Betke-Kleihauer test and flow cytometry discriminate between maternal and fetal red blood cells (RBCs) by utilizing the difference in the chemical properties of fetal and adult hemoglobin in the former, and either the RhD phenotype or the different phenotypes of fetal and adult hemoglobin in the latter. In this context, we note that traditional flow cytometry equipment is highly specialized and expensive and therefore not in general available to practitioners caring for pregnant women. Work by Linderkamp et al. (1983) established that the mean volume of maternal and fetal (in a neonatal setting) RBCs differ significantly, with the average volume of fetal RBCs being 21% greater than that of maternal. Linderkamp et al. (1986a) later showed that the extensional and bending moduli of neonatal RBCs were smaller than for maternal. These differences in size and mechanical properties might under certain setups be utilized as class discriminatory features. We use a previously reported experimental setup with a modified microfluidic chip design (Berg-Sørensen et al. 2019) for flow deformation of fetal and maternal RBCs and capture the dynamic properties by video. To extract the discriminatory features we utilize a deep convolutional neural network (CNN) designed for video recognition, as the expression of the mechanical properties is inherently temporal, and possibly not sufficiently represented in still images.

The potential for using deformation for classifying cells in a flow cytometry setting was first shown by Lincoln et al. (2004). The method was further expanded by Hur et al.  (2011), who demonstrated that the microfluidic properties could directly be used for sorting. The introduction of deformability cytometry in real time (Otto et al. 2015) further demonstrated the strength of microfluidic deformability to distinguish different cells types and was also later applied to cell samples from blood (Toepfner et al. 2018; Kubánková et al. 2021). Other research groups have presented protocols for the construction of such equipment, in a different approach but with comparable characteristics in applicability (Lei et al. 2018). A novelty presented in the present work is the application of injection molded microfluidic chips, ensuring repeatability of microfluidic devices prepared by a production ready method.

Single-cell classification in free flow cytometry has been demonstrated in (Gopakumar et al. 2017) using neural networks. In (Bento et al. 2018) a thorough analysis of different chip designs was performed while (Gu et al. 2019) demonstrated sorting and classification based on fluorescent signals from a microfluidic setting. The so-called real-time deformability cytometry (RT-DC) mentioned above has later been supplemented with fluorescence modalities, surface acoustic waves, and deep neural networks to carry out actual sorting (Nawaz et al. 2020). In (Berg-Sørensen et al. 2019), the potential of using deformation and classification with simple image features was explored. This was later demonstrated in more detail in (Rizzuto et al. 2021), where the authors couple deformation with analysis using a simple neural network. In (Lamoureux et al. 2021) the authors present results for sorting of healthy RBC’s based on deformability using static image data and Deep Learning. Additionally (Eskesen and Friis 2019) showed the efficacy of a single shot detector (SSD) network for RBC classification on still images from the same dataset as that analyzed here. In this work, we expand on the methodology by using a more advanced analysis of the images that directly takes the temporal dimension into account. This is done by utilizing a neural network architecture previously shown to have high accuracy in classifying videos of human activities and applying them to the analysis of RBC deformation.

## Methods

### Experimental assay

**Fig. 1:**
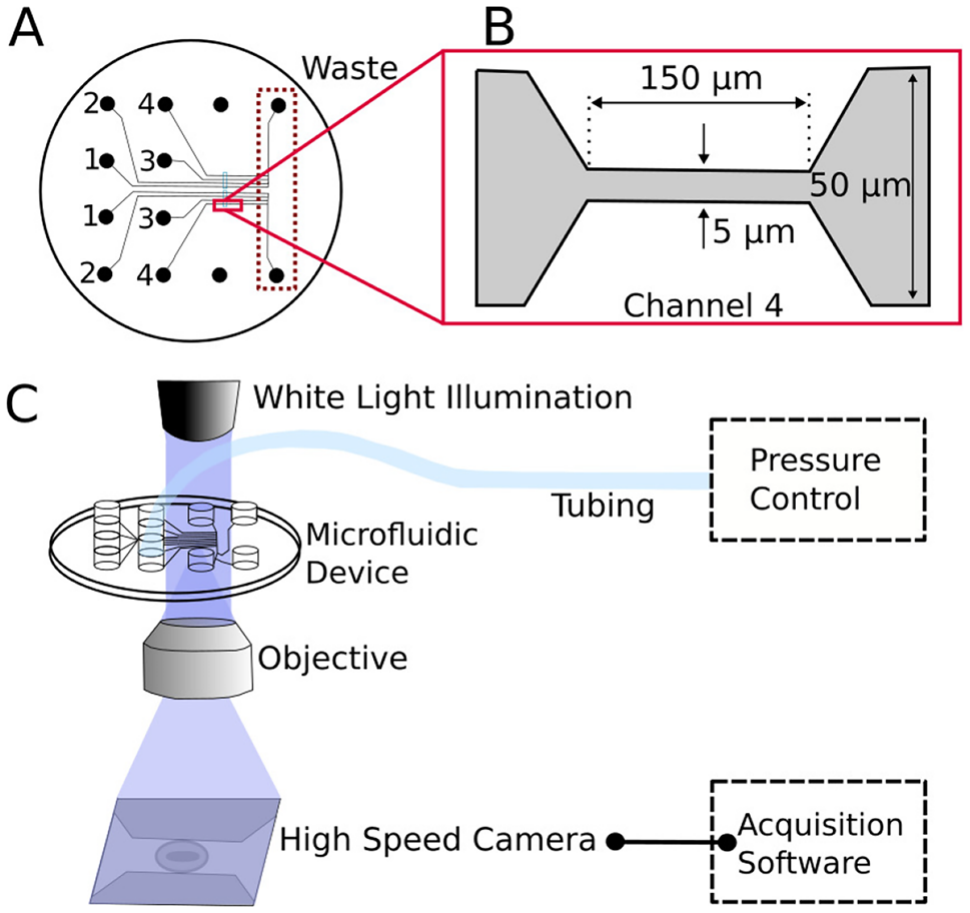
Illustration of the setup for optical video-microscopy of flow in a microfluidic chip. Part **A** provides an overview of the molded microfluidic device with channels for fluidics and for hydrodynamic deformation of the cells. It consist of two sets of four channels (1-4) and two waste outlets. Only the channel of type 4 were used in the experiments reported here. The mirror-design was chosen in order to achieve the most from a preprepared shim for the top part of the chip, containing 12 LUER fittings. Part **B** shows the constricted area in channel 4 with a width of 5µm. This is the place the red blood cells are imaged under flow. Part **C** illustrates the entire setup and how the microfluidic device is placed within a standard bright field inverted optical microscope equipped with a 100x oil immersion objective and a high speed camera with corresponding acquisition software. As explained, the chip is mounted manually using steering pins in a custom-made holder on the microscope. The device is connected to the pressure control and visualized after manual refocusing of the microscope to achieve the best possible image contrast

The experiment is conducted by optical video-microscopy of flow in a microfluidic device. The microfluidic device consists of two sets of four channels (1-4) leading to a common outlet channel. The four channels are designed with different widths of a microfluidic constriction. An overview of the device structure is seen in Fig. 1A. In contrast to the more preliminary work reported previously (Berg-Sørensen et al. 2019), all channels are defined at a single and shallow depth of 10 µm. The altered design was chosen to ensure that the main fraction of individual cells stay within the focal plane when imaged with the optical microscope. The main channels have a cross-section of 50 x 10 µm^2^ while the microfluidic constrictions used in the experiments reported here have a minimum width of 5 µm which was only present in the type 4 channels (Fig. 1B). Other channels of the device have minimum widths of either 10 $\mu$m or 15 $\mu$m. This design allows for the chip to potentially be used with other, larger, cell types. A nickel shim for injection molding was fabricated using standard clean room processes (Utko et al. 2011). The devices were injection molded with the COC polymer TOPAS^®^ grade 5013 following the same procedure as described in (Berg-Sørensen et al. 2019). The microfluidic device is primed with ethanol at high pressure and then filled with an aqueous buffer (CellStab, see details below). Subsequently, 2.6 µl of whole blood is diluted in 1 ml ID-Cellstab^®^ (Biorad, Switzerland), of which 20 µl is added to the LUER well of the microfluidic device. The anonymized whole blood samples are obtained from the blood bank at Rigshospitalet, Copenhagen University Hospital, either as 1 ml samples from donations by regular blood donors giving informed consent for research (whole blood with anti-coagulant EDTA) or as anonymized 1 ml samples from the umbilical cord of delivered placentas, drawn as a standard quality control procedure in the hospital (whole blood with anti-coagulant heparin). A given microfluidic chip has been used for samples from up to 20 donors. Between each new sample, the chip is rinsed thoroughly with CellStab. For each donor the subsequent first analysis provided an average count of 150 cells, giving a gross total of almost 30000 cells investigated.

### Instrument details

The microscope is an inverted Leica DMI 3000B microscope (Leica Microsystems GmbH, Germany) with 100x oil immersion objective (Leica HCX PL FLUOTAR, 100x/1.30), equipped with an AOS S-motion high-speed camera. Recordings were carried out with camera specifications as follows: Frame-rate 500 fps, resolution 800 x 150 pixels, shutter time 25 µs, contrast 8 bit mid. The flow is induced by a Fluigent MFCS-EZ pump (Fluigent, France), from which a pressure of 6 mbar is applied to the microfluidic channel subject to study. With these settings, and blood samples as described above, a throughput of around 2 cells/s was obtained.

For the details of the image- and video-analysis performed, it is important to realize that the microfluidic chips with samples are mounted manually, in a custom-made holder with steering pins, yet not with micrometer accuracy. Similarly, the focusing of the microscope is carried out manually.

An overview of the entire setup is shown in Fig. 1C.

### Data preparation and preprocessing

**Fig. 2:**
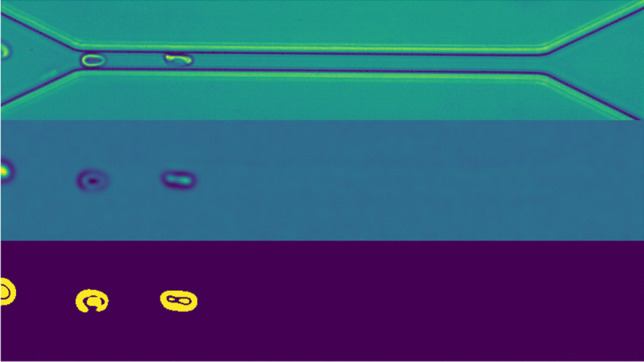
Image of red blood cells. Top image is after application of Gaussian Filter. Middle image is generated by first sampling 100 images from the same donor and using them to calculate the median for each pixel and then subtracting that from the top picture. The bottom image is a binarized version of the middle with threshold set at $|I_{i,j}| > 5$ where *I* denotes the matrix representation of the image

From each sequence (donor), we draw 100 random samples to compute an approximation of a median image. Prior to that, each sample was smoothed with a Gaussian kernel to remove noise. Each image in the sequence was then further smoothed and binarized, by subtracting the median image and subsequently thresholding on the absolute values in the resulting image. Since the channel and background are both temporally invariant, the method yields a binary image with only the RBCs present in the foreground. Specifically we considered $|I_{i,j}| > 5$ as foreground/RBC, where *I* denotes the matrix representation of the grayscale image after median image subtraction. This method proved robust as the subtraction of the image’s own temporal median made it largely invariant to different imaging conditions. We considered each foreground region with a pixel area larger than 50 as representing an RBC. This initial process is visualised in Fig. 2.

**Fig. 3:**
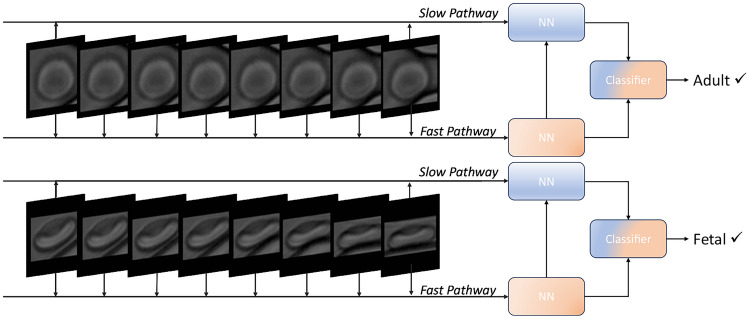
Video data of randomly selected adult RBC (**Top**) and fetal RBC (**Bottom**) in the format fed to the Neural Network. Of the original image of the full channel a box with 25 pixels from all sides of the RBC is cut. Subsequently each image is zero-padded to ensure size conformity. On each of these 8 images we observe the cells starting to enter the inlet and experiencing the first deformation. The figure also shows which frames enter the slow and fast pathway as described in Section 2.4 for classification. Further details of the architecture of the Neural Network are described there as well

From each region, we extract the center and surrounding bounding box. To represent the path of each cell as a video, we first considered the initial image in the sequence. Each RBC presenting region in that image is paired with the region in the subsequent image with the largest shared area. This procedure is carried out for all images in the sequence to produce the subsequences containing RBCs. A video is then captured around the path of each RBC by cropping an area of 50x50 pixels around the center of the RBC, such that we minimize the amount of channel visible through the video. The motivation for this is to prevent the neural network from overfitting on slight variations in the positioning of the channel. The path of an adult and a fetal RBC through the channel subsequent to the preprocessing is seen in Fig. 3. The underlying videos 1 and 2 are available as Supplementary information.

### Neural network

**Fig. 4:**
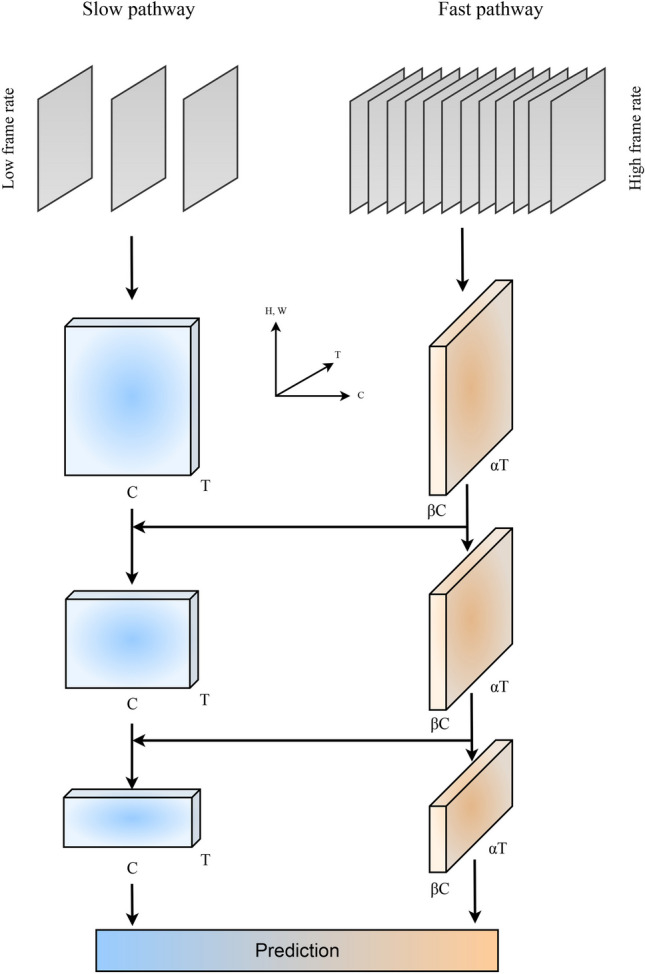
Schematic of the SlowFast CNN architecture. To the right the fast pathway, with connections at each block to the slow pathway (left). The schematic depicts the architecture outlined in (Feichtenhofer et al. 2018) and is based on their depiction

The neural network used in this study was picked due to its explicit handling of the temporal information contained in the image sequences. It was theorized that the deformation of specific blood samples over time was integral to the type of cell, hence validating a focus on the time aspect of the dataset. The SlowFast (Feichtenhofer et al. 2018) architecture applied gets its name from its two pathways. A "Slow" pathway emphasizes the extraction of spatial features from the image sequences, and a "Fast" pathway samples a high temporal resolution input stream to capture motion-related context. A graphical representation of the sentiment behind the architecture can be seen in Fig. 4.

Both pathways are built from 3D ResNet (He et al. 2015) blocks that can handle video input data. In the slow pathway, a temporal stride is defined. It controls the number of frames being processed, meaning the pathway processes only one out of a number of frames. The key concept of the slow pathway is utilizing a large temporal stride, i.e., few of the images of the total image sequence enter the pathway. The focus of the pathway is thus on the spatial structure of the blood cell, capturing non-dynamic features. In this study, $\tau = 8$ is applied, i.e., only every $8^\textit{th}$ image enters the slow path. The fast pathway is the temporal light-weight opposition to the spatial slow pathway. The structure tries to capture movement-related information by processing a far greater fraction of the input images. This fraction is specified by the frame rate ratio, where $\alpha =8$ is used here, i.e. the fast pathway processes 8 images pr. 1 image processed by the slow pathway. This is done to achieve a dense representation along the temporal dimension to capture the red blood cells’ deformation and flow-related information. The number of parameters in the fast pathway is set to be $\beta = \frac{1}{8}$ that of the slow pathway. This is based on the feature space for the topology of the blood cell being substantially more complex than the feature space for the blood cell movement. Throughout the network structure, the information is propagated from the fast to the slow pathway to ensure coherence between the two representations. An illustrative example is provided in Fig. 3. This model was chosen for its high performance on industry-standard video classification datasets and relatively low computational complexity (Feichtenhofer et al. 2018).

### Training

For training, the data was partitioned into 5 disjoint sets of approximately similar size. Since the data consists of several RBCs from each donor we chose to create the splits such that each donor was present in only one set. Four of the sets were allocated as validation sets and one test set. An overview of the allocation of donors and cells at the class level can be seen in Table 1 for the 4 training/validation splits and in Table 2 for the test set. Each split was generated such that the total number of cells (fetal and adult) consisted of approximately 20% of the total number (27226) under the aforementioned restriction on each donor.

**Table 1:** Overview of the allocation of data into the 4 disjoint training/validation sets. The percentages in parenthesis denote the fraction of the total number present across the 5 splits - i.e including the test set. *F* denotes fetal, and *A* adult donor. The number of cells in each set refers to the RBCs taken from the donors present in each split

	Set 1	Set 2	Set 3	Set 4
Num. Donors	32 (19.51%)	34 (20.73%)	32 (19.51%)	34 (20.73%)
Num. Donors (F)	12 (18.46%)	13 (20%)	17 (26.15%)	10 (15.38%)
Num. Donors (A)	20 (20.2%)	21 (21.21%)	15 (15.15%)	24 (24.24%)
Num. Cells	5240 (19.25%)	5613 (20.62%)	5250 (19.28%)	5585 (20.51%)
Num. Cells (F)	1976 (18.64%)	2233 (21.06%)	2707 (25.53%)	1553 (14.65%)
Num. Cells (A)	3264 (19.64%)	3380 (20.33%)	2543 (15.3%)	4032 (24.26%)

**Table 2:** Overview of the allocation of data into the test split. The percentages in parenthesis denote the fraction of the total number present across the 5 splits - i.e including the 4 validation/training splits. *F* denotes fetal, and *A* adult donor

	Test
Num. Donors	32 (19.51%)
Num. Donors (F)	13 (20%)
Num. Donors (A)	19 (19.19%)
Num. Cells	5538 (20.34%)
Num. Cells (F)	2134 (20.13%)
Num. Cells (A)	3404 (20.48%)

Training was carried out with 4-fold cross-validation using early stopping, which means that each model was trained on $\frac{3}{5}$ (3 of the disjoint sets). At every second epoch, the model was evaluated on the fourth disjoint validation set allowing training to terminate once no further improvement on the validation set was observed. An epoch is defined by exposing the network to all training data one time. Since it leads to inefficient training to only make a gradient descent step once the entire training data has been evaluated, we use mini-batch learning with a batch size of 8. We used the SlowFast architecture (Feichtenhofer et al. 2018) with the ResNet 101 depth (He et al. 2015). The network(s) were trained using Stochastic Gradient Descent (SGD) with a learning rate scheme as specified by Loshchilov and Hutter for SGD with Warm Restart (Loshchilov and Hutter 2016). The maximum number of epochs used in the implementation of the learning rate scheme was 250. The maximum number of epochs was observed to be 246 (fold 4), while the minimum was 228 (fold 3), thus fairly close to the predefined maximum. We used the PyTorch implementation of SGD (PyTorch Contributors 2022b) allowing for momentum, chosen to be 0.9. Training also included a dropout rate of 0.5 and was performed on a 12GB Titan XP GPU. As a loss function, we used a cross-entropy loss, a standard choice for binary classification (PyTorch Contributors 2022a). Methods of cropping, flipping, as well as temporal sampling differed at training and inference time. For further details see (Feichtenhofer et al. 2018) (Section 4).

## Results

**Fig. 5:**
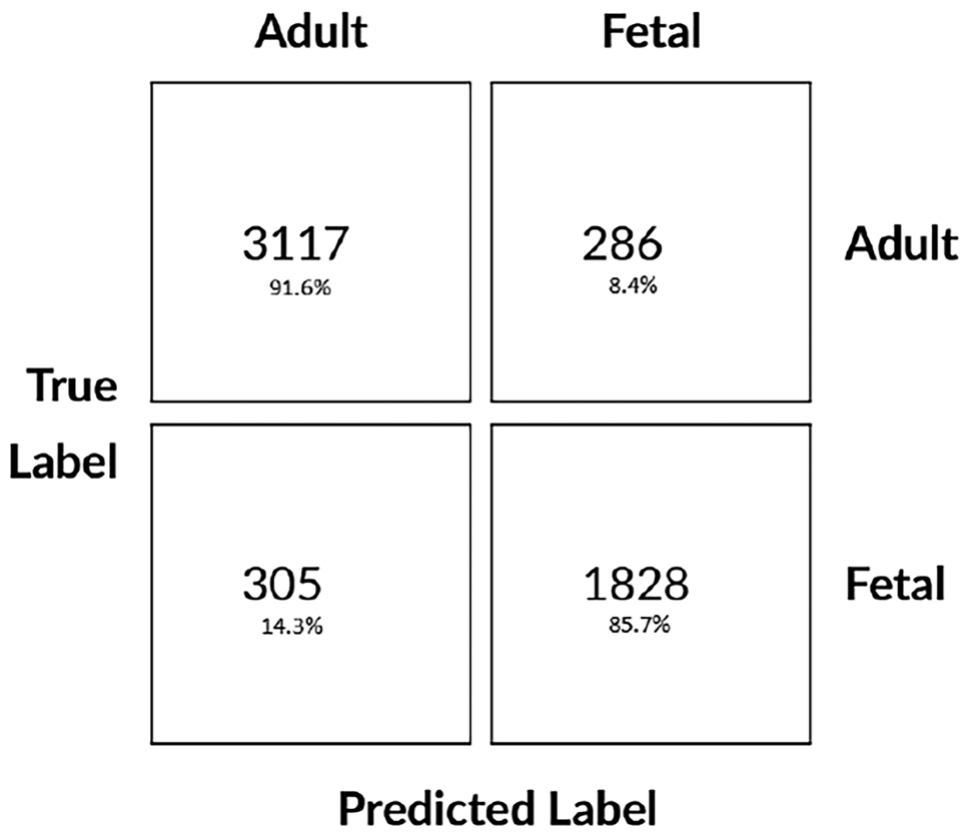
Confusion matrix for evaluation of the 4 networks on the test set. Each entry denotes the mean of that entry across the 4 cross validation folds. The diagonal denotes correct classifications, bottom left denotes misclassifications of fetal cells as adult, and top right denotes misclassifications of adult cells as fetal

In Table 3 we see that the neural network was able to correctly classify the majority of cells with reasonably low deviation in the performance of each cross-validation fold model. The confusion matrix Fig. 5 however, shows that there is some class discrepancy: the model favours the adult RBC’s, which are the most prevalent in the data set. The per class sensitivity (recall) was 91.6% and 85.7% for adult and fetal cells, respectively, further highlighting this class imbalance.

**Table 3:** Summary metrics for overall performance on the test set including 95% confidence intervals. These are calculated under the assumption that each measure follows a normal distribution. AUC = Area Under the Curve

Metric	Score (%)
AUC	90.360 ± 0.330
Sensitivity	88.637 ± 0.366
Accuracy	89.315 ± 0.361
Precision	86.466 ± 0.549
f1	86.072 ± 0.459

### Donor effect

**Fig. 6:**
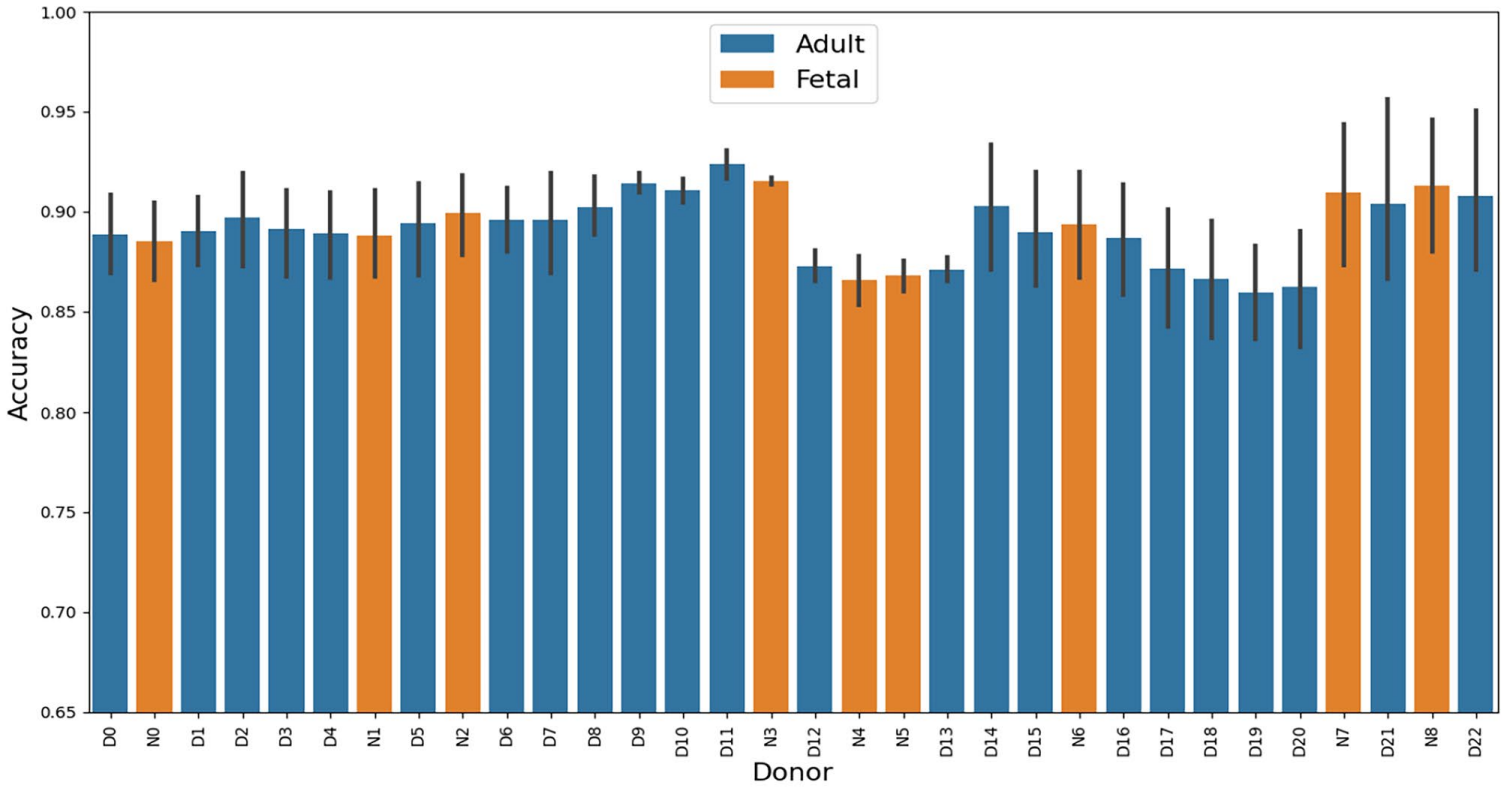
Bar plot over per donor accuracy. Fetal donors are in orange and adult in blue. 95% confidence intervals are indicated with the line on top of each bar and calculated with basis in a normal distribution

We observed some variation between donors, as evident from Fig. 6. The spread equivalently varied between the classes. For adult donors, the highest (average) accuracy was 92.5% with a lowest of 85.9%, thus a difference of 6.4%. For the fetal donors, the highest accuracy was 91.5% with a low of 86.6% and thus a difference of 4.9%, see Table 4. We specifically note that the class performance discrepancy is no longer present when each donor is weighted equally.

**Table 4:** Summary measures of donor specific performance in the test set. First accuracy is calculated for each donor individually by averaging across the 4 networks from each of the cross-validation folds. We report the lowest, highest and combined average of these. Note that this entails that each donor is weighted equally in these summary measures, which is not the case in Table 3

	Lowest (average) accuracy	Mean (donor) accuracy	Highest (average) accuracy
Adult	0.859	0.891	0.925
Fetal	0.866	0.893	0.915

In Table 3 the metrics were calculated without regard to individual donors. Here the accuracy is first calculated for each donor and subsequently averaged. To estimate whether a donor effect was potentially significant, we consider the following Mixed Linear Model

1 $$\begin{aligned} \begin{aligned} A_{d, f}&= \mu + \alpha (D_{d}) + a(F_f) + \epsilon _{d, f}, \\&d \in \{1,\dotsc , 32\}, \ f \in \{1,\dotsc , 4\} \\&a(F) \sim \mathcal {N}(0, \sigma _{A}^2) , \ \epsilon _{d,f} \sim \mathcal {N}(0, \sigma ^2). \end{aligned} \end{aligned}$$

Here, $A_{d,f}$ denotes the accuracy for donor *D* for cross-validation fold *f*. $D_{d}$ denotes the Donors and $\alpha$ the corresponding fixed effect, and *F* denotes the cross-validation folds, included as a random effect *a*. We used the "lmer" function from the R package lmerTest version 3.1-3 (Kuznetsova et al. 2020). The variance of the random effect was not found to be significant $(P = 0.226)$, cf. Table 5. Subsequently the fixed effects part in Eq. 1 was analysed, using the built-in R function for "anova" in R version 4.0.3. The ANOVA table can be seen in Table 6. We note that the donor effect was not found to be significant $(P = 0.853)$. Further, a two-way ANOVA test with results presented in Table 7 substantiates the claim that there is no significant difference (*P* = 0.750) between the performance of the model for the two types of donors.

**Table 5:** ANOVA-like table for random effect *a* in Eq. (1). *NPar* denotes the number of parameters, AIC the Akaike Information Criterion, LRT the likelihood ratio test, Df the number of degrees of freedom and $P(>\chi ^2)$ the p-value. Note here that since variance is only defined for non-negative values, and we are hence testing on the edge of the parameter space, the p-value has been divided by 2

Random Effect	*a*(*F*)
Estimated Variance	0.0000379
NPar	33
Log Likelihood	150.0592
AIC	-234.1184
LRT	0.5662952
Df	1
$P(>\chi ^2)$	0.22585

**Table 6:** ANOVA Table for fixed effects $\mu , \alpha$ in Eq. (1) without the random effect *a*, which was found to not be significant, cf. Table 5. Df denotes the number of degrees of freedom and $P(>F)$ the p-value

	Df	Squared Error	Mean Squared Error	F-Statistic	$P(>F)$
$\alpha (D)$	31	0.03599925	0.001161266	0.7174617	0.8528163
$\epsilon$	96	0.15538328	0.001618576	-	-

**Table 7:** ANOVA Table providing two way ANOVA for test of difference in mean for the per donor performance of the two classes. Note here that $\alpha (M)$ denotes the difference of the adult class from the fetal, that has been subsumed into the intercept. Df denotes the number of degrees of freedom and $P(>F)$ the p-value

	Df	Squared Error	Mean Squared Error	F-Statistic	$P(>F)$
$\alpha (M)$	1	0.0000309	0.00003088	0.1033	0.7501
$\epsilon$	30	0.0089689	0.00029896	-	-

### Estimating data set size implications

**Fig. 7:**
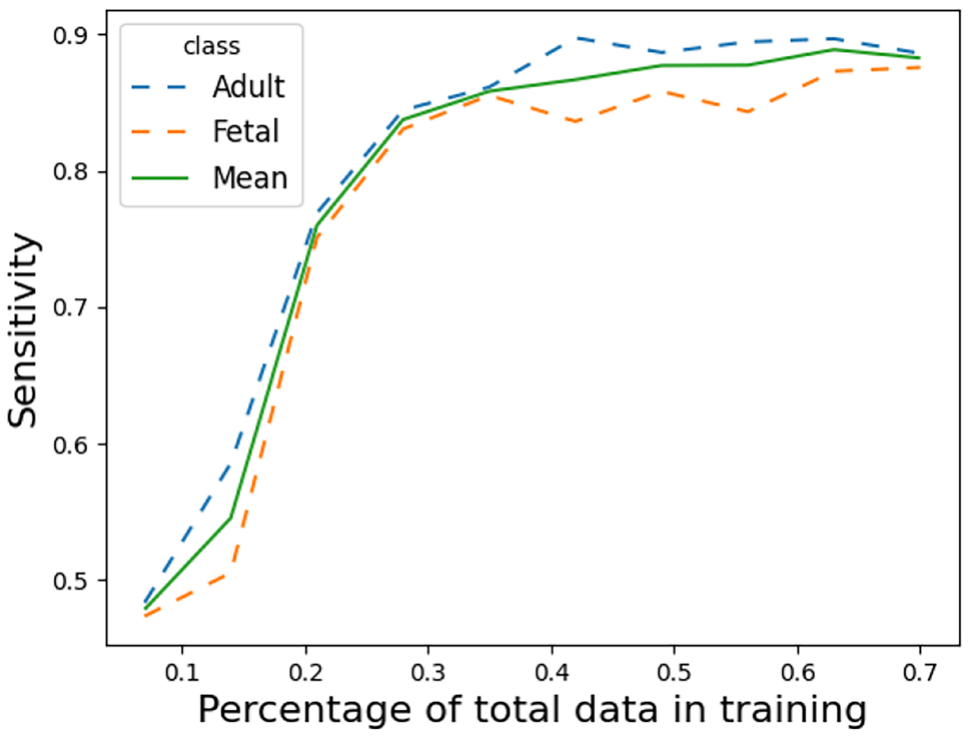
Performance of classifier on the test set as measured by sensitivity, as a function of available data. The performance is measured for single models trained on $\{7\%, 14\%, \dotsc , 70\%\}$ of the total available data. 10% of the data is used to allow for early stopping, while the remaining 20% is allocated in the test set. The mean is calculated by averaging the per class sensitivities

A common drawback of Deep Learning is the necessity for vast amounts of training data. The common notion is that the addition of (quality) data should almost guarantee an improvement in the model’s performance. To estimate the impact of the scale of the data set on this problem, we perform a version of "ramping". Particularly, we seek to understand how the model’s performance scales with the size of the dataset. In order to keep the methodology intact, we allocate 10% of the (training) data as a validation set to allow for early stopping. Subsequently, we train models on $\{10\%, 20\%, \dotsc 100\%\}$ of the remaining training data, where 100% corresponds to 70% of the total data set. Each model can then be evaluated on the test set. The results can be seen in Fig. 7.

It shows that only little improvement is obtained after the inclusion of more than 50% of the total data. We also note a slight decline at 70%. However, both this sensitivity (0.882) and the sensitivity at 60% i.e 0.889 (corresponding to the amount of data used in the cross-validation training) are within the confidence intervals presented in Table 3.

## Discussion

We have presented a method for discriminating between adult and fetal blood cells by exploiting their differences in mechanical properties. We sought to capture these using a deep CNN. First, we showed in Table 3 that the deformation the RBCs experienced through the channel contained a certain amount of discriminatory information achieving an accuracy of 89.315%. However, the performance was not homogeneous between the two classes and does not reach the level required for direct clinical applicability. We showed that the network performed significantly better on adult cells. This discrepancy may be due to the skewed nature of the data set, see Table 1, as adult cells made up 61 % of the total available data.

Additionally we present results in Fig. 7 for simulated states of data availability, under the same global class distribution as in the full data set. These results showed that a limited - and statistically insignificant - gain in performance was achieved after the inclusion of 56% of the data. We therefore propose two interpretations. First, no additional discriminative features were found in the last 14% of the data, i.e., the model would not be able to learn anything relevant from it to increase performance on the test set. This is possibly supported by the low variance of the 4-fold cross validation results presented in Table 3. A second interpretation would be, that a deeper model - a model that contains more parameters - might yield improved results on this data.

We partitioned the data such that each donor was only present in one of the (disjoint) sets. The immediate motivation for this choice was to ensure that the results remained unaffected by potential correlations in the behavior of cells from one donor. It was not the purpose of this study to investigate if there lies some discriminative information within a sample as a whole. This means that we did not analyze whether a donor - or groupings within the set of donors, other than fetal and adult - could be identified by the deformations of the RBCs. Such correlation structures could also arise from flaws in the experimental setup. Thus, the choice of making the sets disjoint in this way was also highly motivated by the possibility of limiting the influence of such flaws. The final motivation for this choice was to better approximate how this method would perform in practice where a new patient could not possibly be present in the data set prior to inference. Furthermore, including each donor in training would severely limit the practical applications of this method.

Finally we shall comment on the data representation. Fetal and maternal RBCs differ in both size and deformative moduli (Linderkamp et al. 1986a). These are only fully represented in 3 dimensions since an RBC is not symmetric over all axes. In this work, we represent the structure in a shallow channel in 2-dimensional space where a considerable fraction of the cell volume is within the focal depth of the imaging objective. Still, certain discriminatory information present in 3 dimensions may not remain present in the projection onto 2 dimensions. Essentially, this means that certain deformations of the RBC may not be visible from the angle we observe from. For the temporal side of the data representation we note, that previous attempts at using still images from the same dataset for RBC classification (Eskesen and Friis 2019), yielded a mean accuracy of 70.18 %. Compared to the results presented in Table 3 we show that the inclusion of temporal information provides a significant performance increase.

### Supplementary Information

Below is the link to the electronic supplementary material.

Supplementary file 1 (avi 876 KB)

Supplementary file 2 (avi 251 KB)

## Data Availability

Data are available from corresponding author upon reasonable request.
